# Palliative care impact on hospital utilization and trends among lung cancer admissions with a high risk of mortality in U.S. hospitals

**DOI:** 10.3389/frhs.2026.1801511

**Published:** 2026-07-13

**Authors:** Jinwook Hwang, Yonsu Kim, David Picella, Sanggon Nam, Diane Chau, Diana Woods, Jongho Won, Iulia Ioanitoaia-Chaudhry, Ronald Tan, David Byun, Maryam Tabrizi, Isabelle Kwak, Ji Won Yoo

**Affiliations:** 1 Korea University Ansan Hospital, Ansan-si, Republic of Korea; 2 University of Nevada Las Vegas, Las Vegas, NV, United States; 3 Azusa Pacific University, Azusa, CA, United States; 4 VA Long Beach Healthcare System, Long Beach, CA, United States; 5 Hongik University, Mapo-gu, Republic of Korea; 6 VA Southern Nevada Healthcare System, Las Vegas, NV, United States; 7 William Bee Ririe Rural Hospital and Clinics, Ely, NV, United States; 8 School of Dental Medicine, University of Nevada Las Vegas, Las Vegas, NV, United States; 9 The Connection Sphere, Las Vegas, NV, United States; 10 Kirk Kerkorian School of Medicine, University of Nevada Las Vegas, Las Vegas, NV, United States

**Keywords:** 2016, high risk mortality, hospital, lung cancer, palliative care

## Abstract

**Background:**

The top 5% of healthcare utilization accounts for 60% of healthcare expenditure in the U.S., with the majority of the costs occurring in the last year of life for hospital inpatient services for diseases such as lung cancer (LC). Previous years' concentrated healthcare expenditure burdens have been shared globally. The research questions of this study are whether palliative care (PC) is associated with hospital utilization and what the PC trends are in U.S. hospitals. Thus, this study aimed to examine (1) the impact of hospital PC on hospital utilization charges and length of stay (LOS), and (2) PC trends, particularly before and after 2016 when a tri-convergent shift occurred in federal reimbursement, professional clinical guideline establishment, and legislative policy.

**Methods:**

This was a retrospective study that analyzed the National Inpatient Sample between 2002 and 2021. Adults 18+ years with LC and high-risk mortality were identified as the denominator. PC utilization was the main outcome measurement and the numerator. Hospital LOS (in days) and charges were secondary outcome measurements. An interrupted time series analysis was applied in 2016 as a U.S. healthcare breakpoint.

**Results:**

A total of 11,185,408 admissions were identified. PC utilization increased from 2.38% in 2002–2006 to 7.74% in 2007–2011, 13.29% in 2012–2016, and 15.86% in 2017–2021 (*p* < 0.001). The upward trend in PC utilization has decelerated by 6.40% (*t* = −9.67, *p* < 0.001) since 2016. PC was associated with a 1.22-day shorter LOS (*t* = −48.2, *p* < 0.001) and hospital charges that were $13,208.85 lower (*t* = −39.95, *p* < 0.001).

**Conclusion:**

PC utilization increased but slowed from 2016, with underuse persisting (<20% in 2021) among LC admissions in U.S. hospitals. PC utilization improves efficiency among patients with LC and a high risk of mortality by reducing hospital LOS and charges by 15%, respectively.

## Introduction

In 2026, 124,990 individuals are estimated to die from lung cancer (LC) in the U.S. This will account for 20.2% of all cancer deaths, making it the leading cause of cancer-related death among both men and women (1). Although palliative care (PC) benefits for those with terminal LC have been published elsewhere (2–4), the transition from life-sustaining treatment (LST) to PC remains late, and more LST has been administered over the past decade (2, 3). As acuity scores and ventilator utilization have been gradually increasing in critical care over the past decades (5), especially during the COVID-19 pandemic era (6), concentrated healthcare expenditure is a major public burden in the U.S. The top 5% of healthcare utilization accounts for 60% of healthcare expenditure in the U.S. (7), with the majority of the costs occurring in the last year of life for hospital inpatient services (8, 9). One quarter of all Medicare spending goes toward care for people during their last year of life (7). The Medicare reimbursement burden has escalated as LST, for example, mechanical ventilation (MV), and late referral to PC occur in those with LC in U.S. hospitals (9). Many hospitalized adults with cancer in U.S. hospitals are seriously ill and remain at high risk of death (2–4, 10). Risk of mortality, as defined by the All-Patient Refined Diagnosis-Related Group (APR-DRG) classification system developed by 3M Health Information Systems, has distinct, categorical subclasses representing the likelihood of in-hospital mortality and is categorized into four ordinal levels (subclass 1, minor risk of mortality; subclass 2, moderate risk of mortality; subclass 3, major risk of mortality; and subclass 4, extreme risk of mortality) (11). Ideally, access to PC is provided to all cancer admissions with a risk of mortality or at least to those in subclasses 3 and 4. LC patients with a high risk of mortality, who often have complex comorbidities and intensive hospitalizations (12, 13), rely on PC to align treatments with their values, reducing futile interventions (3, 4). For non-cancer patients with a high risk of mortality, early referral to PC is also suggested to increase care efficiency, particularly in inpatient care settings (14).

During the past decade, the U.S. healthcare system has undergone policy changes related to both patients with LC and inpatient PC services. We identified 2016 as a U.S. healthcare structural policy breakpoint based on a tri-convergent shift in federal reimbursement, national clinical guidelines, and legislative policy. Specifically, January 2016 marked the implementation of the Centers for Medicare and Medicaid Services (CMS) reimbursement for advance care planning (ACP), providing the financial infrastructure for inpatient goals-of-care discussions (15). Concurrently, the American Society of Clinical Oncology (ASCO) issued its landmark 2016 guideline mandate, reinforcing early, universal concurrent PC for patients with advanced LC (16). Finally, selecting 2016 provides a stable baseline following the October 2015 ICD-10 transition, minimizing coding bias (17).

Little is known about the PC trend and its impact on hospital utilization among patients with LC and a high risk of mortality. The research questions of this study are whether PC is associated with hospital utilization, and what the PC trend was before and after 2016 in U.S. hospitals. Using nationally representative hospital claim data from two decades, we aimed to examine (1) the impact of hospital PC on hospital utilization, i.e., hospital charges and length of stay (LOS), and (2) the PC trend among patients with LC and a high risk of mortality, particularly before and after 2016, when a tri-convergent shift occurred in federal reimbursement, professional clinical guideline establishment, and legislative policy.

## Methods

### Data source and study procedure

A serial, cross-sectional retrospective analysis of the National Inpatient Sample (NIS) dataset was performed. The NIS is a dataset from the Healthcare Charge and Utilization Project (HCUP) and includes an approximately 20% stratified sample of discharges representing more than 97% of U.S. community hospitals from more than 40 states (18). The NIS dataset contains deidentified and random samples of hospitalizations recorded by HCUP member hospitals. This information was also stratified by hospital location and teaching status as indicated on the American Hospital Association Annual Survey (18). Because the NIS dataset is a large secondary dataset, upon completion of a data user agreement with the Agency for Healthcare Research and Quality (AHRQ), the University of Nevada, Las Vegas (UNLV) Institutional Review Board (IRB) found that the current study was exempt (UNLV-2025-684). Thus*,* consent to participate was waived by the UNLV IRB. This study was not a clinical trial. The data were accessed for research purposes between 28 November 2025 and 19 May 2026.

### Study subjects' selection

Adults 18 years and older with a primary diagnosis of LC, a high risk of mortality, and admitted from 2002 to 2021 were identified. LC was defined as International Classification of Diseases, 9th revision, Clinical Modification (ICD-9-CM) codes 162.0, 162.2, 162.3, 162.4, 162.6, 162.8, and 162.9 and ICD-10 code C34 (10, 19). A high risk of mortality was defined as the All-Patient Refined Diagnosis-Related Group (APR-DRG) risk of mortality subclass 3 or 4. The four subclasses are as follows: 1, no/minor; 2, moderate; 3, major; 4, extreme complications (2, 11–14). Approximately 60% of all cases with primary diagnoses of cancer were admitted with a high risk of mortality [APR-DRG 3 (major) or 4 (extreme) complications] in U.S hospitals (2, 11–13). The NIS data for 2002–2011 include both hospital and discharge weights. The hospital weights estimate hospital-level values, and the discharge weights estimate discharge-level values. In the NIS dataset, from 2012 onwards, only discharge-level values were weighted (18). Therefore, the change in 2012 did not affect our estimate of PC utilization trends.

### Variables and outcome measurements

The sociodemographic characteristics recorded for each hospital admission included age, sex, race/ethnicity (White, Black, Hispanic, Asian/Pacific Islander, or other), and payer source [government payers (Medicare and Medicaid), private insurance, or other] (2, 12–14). MV was selected as an example of a common LST procedure for patients with LC and a high risk of mortality (2, 20). MV was identified using ICD-9-CM code 39.65 or ICD-10-CM codes 5A1935Z, 5A1945Z, or 5A1955Z (20). PC utilization, LOS, and hospital charges were selected as healthcare utilization factors. PC utilization was identified using ICD-9-CM code V66.7 and ICD-10 code Z51.5. These codes have been validated as the optimal method for identifying inpatient PC services in previous studies (2, 14, 19). Yearly hospital charges were adjusted by applying the Bureau of Labor Statistics' Consumer Price Index (CPI) for Medical Care (21). Among the utilization factors, PC utilization was chosen as the primary outcome. LOS and hospital charges were the secondary outcomes.

### Statistical analysis and appraisal checklist

First, we performed trend analyses for each variable and outcome in each 5-year period, i.e., 2002–2006, 2007–2011, 2012–2016, and 2017–2021. Analysis of variance was used for continuous variables (age, LOS, and hospital charges) and Pearson’s *χ*^2^ test was used for categorical variables (age group, sex, race/ethnicity, payer source, MV, and PC utilization) to assess the statistical significance of the trends for each variable and outcome. Second, an interrupted time series (ITS) analysis was performed to examine the temporal associations with PC utilization. The ITS analysis has been applied to quantify the impact of a health policy intervention at the population level over a defined enactment timeline (22, 23). We created an aggregated time series variable with a monthly unit and this procedure created a total of 320 (12 months per year × 20 years) time series for PC utilization per LC admission with a high risk of mortality. In addition, a multivariate regression model was applied to the ITS analysis by controlling for the demographic characteristics, payer source, and MV utilization variables. As a sensitivity analysis, total hospital admission was treated as the denominator (dependent variable) and PC utilization was used as the numerator (independent variable in the regression model). Finally, we performed multivariate regression analyses by treating LOS and hospital charges as dependent variables. We stratified these multivariate regression analyses into before and after 2016, a policy-relevant temporal marker. Two-sided *p*-values were reported and *p* < 0.05 was considered statistically significant. SAS version 9.4 (SAS Institute, Cary, NC, USA) was used for all statistical analyses. The randomness of missing values was assessed by applying multiple imputation with chained equations (24). The absence of missing value-related randomness was confirmed for all variables (*p* < 0.05). The Joanna Briggs Institute (JBI) critical appraisal checklist was applied in this analytical observational study, and is presented in the Supplementary Table.

## Results

A total of 11,185,408 admissions were identified. Table 1 presents descriptive statistics and trend analysis for each variable in each 5-year period.

**Table 1:** Descriptive analysis and statistics for each variable.

	Years	2002–2006		2007–2011		2012–2016		2017–2021			2002–2021	
		*n*, mean	%, *SD	*n*, mean	%, *SD	*n*, mean	%, *SD	*n*, mean	%, *SD	*p*	*N*, mean	%, *SD
Total, *n*		172,571		214,974		237,374		256,009			11,185,408	
Sex	Female	76,305	44.22	97,557	45.38	110,663	46.62	121,370	47.41	<0.001	405,895	46.08
	Male	96,266	55.78	117,417	54.62	126,711	53.38	134,639	52.59		475,033	53.92
Age		69.71	11.12	69.98	11.13	68.98	10.73	69.07	10.32	<0.001	69.14	10.80
Age group	19–35	326	0.19	442	0.21	447	0.19	472	0.18	<0.001	1,687	0.19
	36–54	19,650	11.39	23,559	10.96	22,402	9.44	17,207	6.72		82,818	9.40
	55–64	37,758	21.88	47,213	21.96	56,163	23.66	61,171	23.89		202,305	22.96
	≥65	114,837	66.54	143,760	66.87	158,362	66.71	177,159	69.20		594,118	67.44
Race/ethnicity	White	96,758	56.07	141,693	65.91	176,164	74.21	191,960	74.98	<0.001	606,575	68.86
	Black	15,446	8.95	23,866	11.10	29,576	12.46	33,001	12.89		101,889	11.57
	Hispanic	4,880	2.83	6,536	3.04	9,824	4.14	11,565	4.52		32,805	3.72
	Asian/Pacific Islander	2,879	1.67	4,439	2.06	5,617	2.37	7,389	2.89		20,324	2.31
	Other	52,608	30.48	38,440	17.88	16,193	6.82	12,094	4.72		119,335	13.55
Pay source	Government	126,368	73.23	157,755	73.38	180,876	76.2	198,879	77.68	<0.001	663,878	75.36
	Private	38,833	22.5	47,293	22.00	45,316	19.09	45,012	17.58		176,454	20.03
	Other	7,370	4.27	9,926	4.62	11,182	4.71	12,118	4.73		40,596	4.61
Mechanical ventilation (yes)		17,394	10.08	19,653	9.14	20,445	8.61	20,424	7.98	<0.001	77,916	8.84
Outcome measurements												
Palliative care (yes)		4,110	2.38	16,643	7.74	31,554	13.29	40,607	15.86	<0.001	92,914	10.55
Length of stay (days)		9.07	9.04	7.83	7.58	7.14	6.64	7.05	6.80	<0.001	7.66	7.48
Hospital charges ($)		68,113	91,395	73,355	96,045	76,585	96,467	85,418	111,089	<0.001	76,737	100,146

SD, standard deviation for age, length of stay, and total charge variables.

The proportion of female patients increased from 44.22% in 2002–2006, to 45.38% in 2007–2011, 46.63% in 2012–2016, and 47.41% in 2017–2021 (*p* < 0.001). The mean ± standard deviation (SD) age decreased from 69.71 ± 11.12 years in 2002–2006 to 69.07 ± 10.32 years in 2017–2021 (*p* < 0.001). The 65 years and older age group accounted for two-thirds of total admissions, i.e., 66.54%, in 2002–2006 and 69.20% in 2017–2021. The proportion of White patients increased from 56.07% in 2002–2006 to 65.91% in 2007–2011, 74.33% in 2012–2016, and 74.98% in 2017–2021 (*p* < 0.001). The proportion of patients categorized as “Other race/ethnicity” decreased from 30.48% in 2002–2006 to 4.72% in 2017–2021 (*p* <0 .001). The proportion of those paying using government sources increased from 73.23% in 2002–2006 to 73.38% in 2007–2011, 76.38% in 2012–2016, and 77.68% in 2017–2021 (*p* < 0.001). MV utilization decreased from 10.08% in 2002–2006 to 7.98% in 2017–2021 (*p* < 0.001). PC utilization increased from 2.38% in 2002–2006 to 7.74% in 2007–2011, 13.57% in 2012–2016, and 15.86% in 2017–2021 (*p* < 0.001). LOS decreased from a mean (SD) of 9.07 (9.04) days in 2002–2026 to 7.83 (7.58) days in 2007–2011, 7.11 (6.63) days in 2012–2016, and 7.05 (6.80) days in 2017–2021 (*p* < 0.001). The mean (SD) hospital charges increased from $40,711 ($54,592) in 2002–2006 to $52,850 ($69,383) in 2007–2011, $64,426 ($81,181) in 2012–2016, and $81,623 ($106,713) in 2017–2021 (*p* < 0.001).

**Figure 1:**
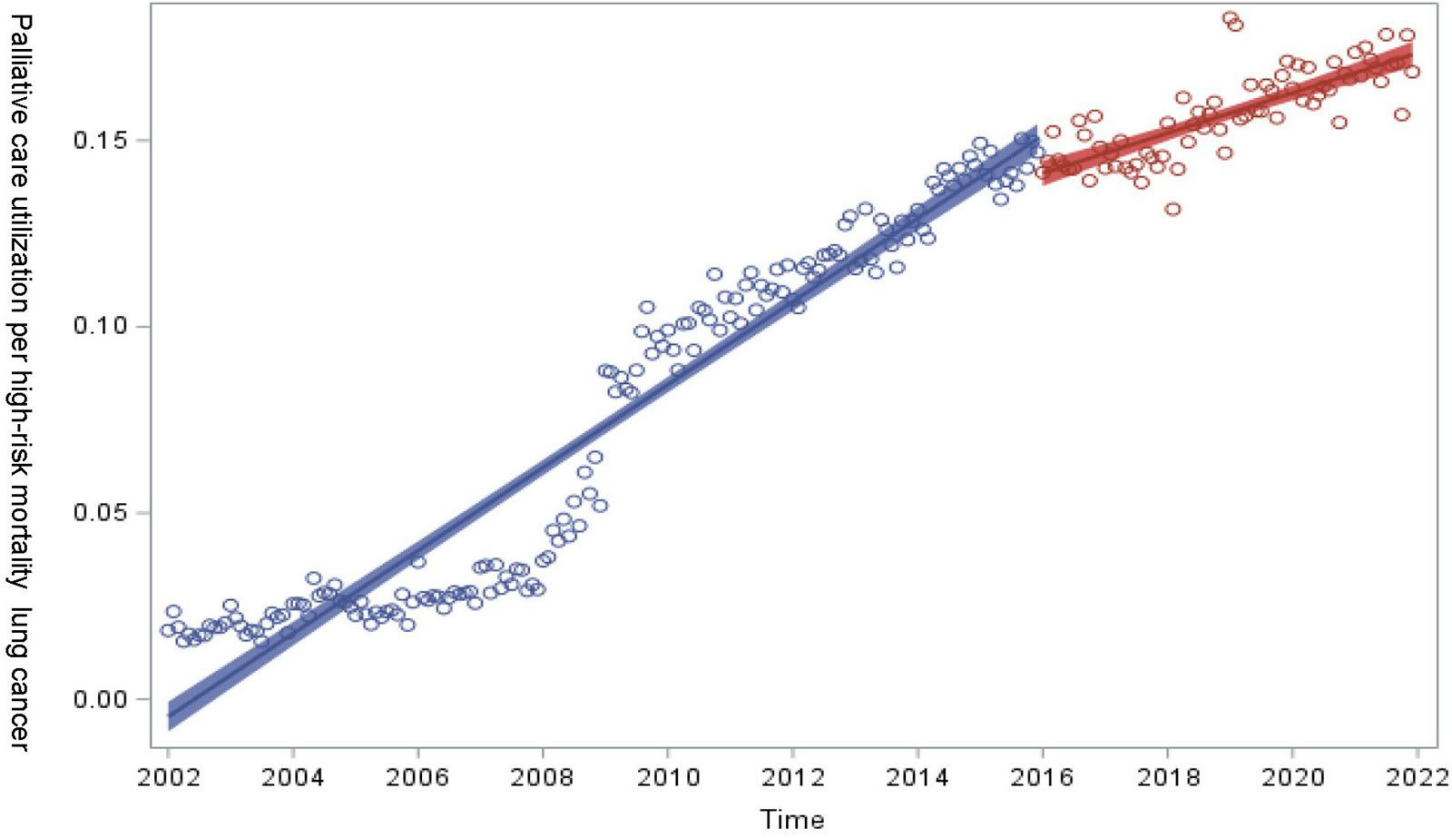
Interrupted time series of palliative care utilization among high-risk mortality lung cancer admission.

The upward trend in PC utilization slowed by 6.40% (*t* = −9.67, *p* < 0.001) after 2016, as illustrated in Figure 1. These results were unchanged after a sensitivity analysis was performed using total hospital admissions as the denominator (dependent variable). Tables 2 and 3 presents the multivariate regression analysis results of the impact of PC utilization on LOS and hospital charges. PC utilization was associated with a 1.22-day shorter LOS (*t* = −48.20, *p* < 0.001) and hospital charges that were lower by $13,208.85 (*t* = −39.95, *p* < 0.001). Compared to the referent age group of 65 years and older, all the younger age groups, i.e., 19–35, 36–54, and 55–64, were associated with shorter hospital LOS (*p* < 0.001). Being female was associated with a prolonged hospital LOS by 0.10 days (*t* = 6.54, *p* < 0.001). Compared to White patients, identifying as a racial/ethnic minority was associated with prolonged hospital LOS, increased by 0.89 days for Black patients (*t* = 36.06, *p* < 0.001), 0.68 days for Hispanic patients (*t* = 16.66, *p* < 0.001), 0.76 days for Asian/Pacific Islander patients (*t* = 14.74, *p* < 0.001), and 0.43 days for those who were categorized as “Other” (*t* = 18.97, *p* < 0.001). Compared to government payment source, private insurance and other pay sources were associated with shorter hospital LOSs, by 0.23 (*t* = −10.60, *p* < 0.001) and 0.24 days (*t* = −0.24; *p* < 0.001), respectively. Compared to APR-DRG subclass 3, APR-DRG subclass 4 was associated with a hospital LOS prolonged by 2.89 days (*t* = 150.30, *p* < 0.001). MV utilization was associated with a hospital LOS prolonged by 2.96 days (*t* = 99.3, *p* < 0.001).

**Table 2:** Multivariate regression analysis of the impact of palliative care utilization on hospital length of stay.

Parameter	Referent	Estimate (days)	*t*	*p*
Intercept		8.20		
Palliative care	None	−1.22	−48.20	<0.001
Female	Male	0.10	6.54	<0.001
Age	65 years and older			
19–35 years		−1.29	−7.25	<0.001
36–54 years		−1.50	−8.51	<0.001
55–64 years		−1.74	−9.83	<0.001
Race/ethnicity	White			
Black		0.89	36.06	<0.001
Hispanic		0.68	16.66	<0.001
Asian/Pacific Islander		0.76	14.74	<0.001
Other		0.43	18.97	<0.001
Pay source	Government			
Private insurance		−0.23	−10.60	<0.001
Other		−0.24	−6.52	<0.001
Mechanical ventilation	None	2.96	99.30	<0.001
APR-DRG	Subclass 3			
Subclass 4		2.89	150.30	<0.001

APR-DRG, All-Patient Refined Diagnosis-Related Group.

**Table 3:** Multivariate regression analysis of the impact of palliative care utilization on hospital charges.

Parameter	Referent	Estimate ($)	*t*	*p*
Intercept		91,096.31		
Palliative care	None	−13,208.85	−39.95	<0.001
Female	Male	−708.71	−3.50	<0.001
Age	65 years and older			
19–35 years		−28,371.77	−12.16	<0.001
36–54 years		−28,962.94	−12.48	<0.001
55–64 years		−32,640.61	−14.08	<0.001
Race/ethnicity	White			
Black		5,549.08	17.22	<0.001
Hispanic		29,353.46	54.51	<0.001
Asian/Pacific Islander		38,054.18	55.66	<0.001
Other		−9,931.68	−33.18	<0.001
Pay source	Government			
Private insurance		1,250.93	4.42	<0.001
Other		−5,308.28	−10.72	<0.001
Mechanical ventilation	None	79,388.88	204.03	<0.001
APR-DRG	Subclass 3			
Subclass 4		39,397.92	156.96	<0.001

APR-DRG, All-Patient Refined Diagnosis-Related Group.

In terms of hospital charges, the 19–35, 36–54, and 55–64 age groups were associated with lower hospital charges (*p* < 0.001). Being female was associated with a $708.71 lower hospital charge (*t* = −3.50, *p* < 0.001). Compared to White patients, Black, Hispanic, and Asian/Pacific Islander patients were associated with higher hospital charges, increased by $5,549.08 (*t* = 17.22, *p* < 0.001), $29,353.46 (*t* = 54.51, *p* < 0.001), and $38,054 (*t* = 55.66, *p* < 0.001), respectively. Being categorized as “Other” was associated with a $9,931.68 lower hospital charge (*t* = −33.18, *p* < 0.001). Compared to government payment, private insurance was associated with a $1,250.93 higher hospital charge (*t* = 4.42, *p* < 0.001); having an “other” payment source was associated with a $5,308.28 lower hospital charge (*t* = −10.72, *p* < 0.001). Compared to APR-DRG subclass 3, APR-DRG subclass 4 was associated with a $39,397.92 higher hospital charge (*t* = 156.96, *p* < 0.001). MV utilization was associated with a $78,338.88 higher hospital charge (*t* = 204.03, *p* < 0.001).

## Discussion

This study answered the following research questions: whether PC is associated with hospital utilization and what the PC trend was before and after 2016 in U.S. hospitals. To the best of our knowledge, this study is the first examination of the association between PC utilization and hospital utilization among LC admissions with a high risk of mortality over two decades. PC utilization increased over this period, but the increasing trend has slowed after 2016. The potential benefits of hospital PC, demonstrated by the associations with reductions in both hospital LOS and charges by approximately 15% (1.22 days per a total LOS of 8.20 days; $13,208 per a total hospital charge of $91,096, respectively), were identified in this study. The benefits of PC utilization in this study are aligned with an increase in healthcare utilization efficiency through the mitigation of the burden on the CMS to reimburse often non-beneficial LST in patients with terminal LC in acute care settings (8, 9). Although PC utilization has been trending upwards, PC utilization remained underutilized in our study. PC utilization in the most recent observation year, 2021, was less than 20%. The increasing trend in PC utilization in our study was mirrored by the finding of a decreasing trend in MV utilization. As MV utilization was associated with higher hospital charges ($79,388.88) and longer hospital LOS (2.96 days), the choice between PC and MV utilization in our study population was rather discrete, as noted in previous studies (2, 7, 9, 14).

The observed 25.4% increase in the mean hospital charge in this study reflects broader, systemic trends in the healthcare economy over the 20-year analysis window. First, this increase was heavily driven by the rapid adoption of highly specialized medical technology, sophisticated diagnostics, and high-value therapeutics, which dramatically increase the baseline cost and structural complexity of standard inpatient care (25). Second, the period between 2002 and 2021 saw immense hospital market consolidation across the U.S. (26). This consolidation reduced local competition and expanded health system asset concentration, which empirically correlates with an increase in healthcare charges and commercial pricing power (26). Finally, demand-side factors—primarily an aging demographic burdened by a higher prevalence of complex, chronic conditions—have escalated the clinical intensity and resource allocation required per average hospital LOS.

The historical increase in the proportion of women in this LC cohort correlates with a well-documented epidemiological shift: a rising incidence of LC in women, particularly involving advanced-stage diagnoses and specific cancer types (such as adenocarcinoma) over time. Overall, the LC age-standardized incidence rates have sharply and quickly increased in women compared to men in recent decades (1, 27). LC is associated with a significant financial burden globally. In 2024, LC was responsible for 1.8 million deaths and was the leading cause of death among all cancers (28). Managed care frameworks and national health benefit packages should expand their coverage for LC screening to non-smokers and increase access to PC in home-based and outpatient care settings. Shifting the institutional setting away from high-tariff inpatient hospital stays toward home-based or outpatient support directly reduces terminal-phase or late-referred PC services.

Inpatient PC utilization and ACP establishment at ambulatory care centers cannot be discussed separately in real-world practice. Increased ACP establishment at ambulatory care centers may be associated with a reduction in unnecessary hospital admissions (29). An annual wellness visit (AWV) has been highlighted as an opportunity for Medicare beneficiaries to be exposed to, discuss, and document ACP, regardless of illness type and stage (30, 31). To increase PC uptake, several strategies have been considered and applied to targeted populations and clinical settings. Through the geriatric workforce enhancement programs, interprofessional education with simulation training has enhanced the skills and experiences of primary care providers' discussion and documentation of ACP (32–34). Along with this effective workforce education, ACP billing reimbursement to ambulatory healthcare providers has triggered synergistic effects in healthcare providers, health systems, and community stakeholders, advancing care efficiency for individuals with complex medical illnesses, including advanced cancers (33, 34). Disseminating the above benefits of ACP establishment through interprofessional education with simulation training was found to be more effective among ethnic minorities and in underserved communities (31, 33). Increasing awareness and access is also a proven community-based delivery model in rural communities (33, 35). Traditional clinical consultations are often heavily compressed, leaving physicians with inadequate time to address complex social and anticipatory care needs (33, 35). Health fairs offer an unstructured, experiential learning environment (32, 34). This allows interprofessional teams—comprising peers and healthcare professionals—to dedicate the necessary time to guide individuals through sensitive discussions without the rush of a typical clinical appointment (33, 35). Choosing PC utilization in inpatient care settings, especially for those with a high risk of mortality, such as those included in this study, raises various decision-making challenges (12). Racial/ethnic minorities within this population have limited awareness and knowledge of ACP and utilize less PC compared to non-Hispanic White patients (33, 34, 36). This limited awareness and knowledge of ACP and late referral to PC among racial/ethnic minorities may lead to delayed decision-making in shifting from life-sustaining treatment to PC in hospitals (37). This delayed decision-making likely prolonged hospital LOS and led to higher hospital charges in this study. This process can be applied to other findings, such as interpretations of the impact of high acuity conditions, APR-DRG subclass 4, and MV utilization on hospital LOS and charges. Thus, the impact of CMS reimbursement for ACP extends to all stakeholders in the care of Medicare beneficiaries. To disseminate ACP discussions more effectively, workforce training in primary care is more practical compared to structural investment (i.e., increase of hospital beds), especially in workforces serving racial minorities and rural residents who underutilize PC. Targeted ACP training, telehealth outreach, and community partnerships can improve access for underserved populations.

As the first-billed ACP of individuals with a high risk of mortality shifted from inpatient to ambulatory and AWV care settings, the shift in sites of death from inpatient care to postacute care or hospice service delivery may be associated with this shift to ACP-billed settings (38–40). The establishment of ACP has further implications for advancing care efficiency in the midst of a payment system shift from traditional episode-based payment to value-based payment. For example, bundled payment systems promote better collaboration and care coordination between healthcare providers (40).

The results of this study should be cautiously interpreted due to it being a secondary analysis of administrative and claim databases. The relationship between PC and ACP was not tested within the dataset and therefore, this remains speculative. This relationship cannot be inferred causally from the present study design because deidentified admissions were analyzed without identifying multiple admissions as the same individual. The selection of PC codes instead of do-not-resuscitate (DNR) and ACP codes was justified because the frequencies of DNR and/or ACP codes were only approximately 20% of the frequency of PC codes and the validity of the PC codes has been well-documented elsewhere (2, 17, 19). PC use may be underreported due to provider barriers such as time constraints or inconsistent documentation, potentially leading this study to underestimate its impact. In terms of clinical perspectives, the choice of PC codes is optimal for collecting cases in transition from life-sustaining management to hospice care. The coding system transition from ICD-9 to ICD-10 occurred on 1 October 2015 (17). This transition may have led to possible under-documentation across all diagnoses and procedures (17). NIS collects each discharge without personal identification; thus, readmission was not counted in this study. We could not differentiate between LC stages and instead, only the primary diagnosis of LC was selected to represent acuity and to assure the homogeneity of the analysis sample. The APR-DRG system was applied to indicate the medical complexity of the study cohort. The proportion of “Other race/ethnicity” in 2002–2006 was remarkably high at 30.48%. However, our ITS and regression analysis results were unchanged with or without the “Other race/ethnicity” cohort. The year 2016 should be cautiously interpreted as a policy-relevant temporal marker to examine the temporal association with PC utilization using ITS, as it does not examine causal attribution.

## Conclusion

PC utilization increased but slowed from 2016, with underuse persisting (<20% in 2021), among LC admissions in U.S. hospitals. PC utilization improves efficiency among patients with LC and a high risk of mortality by reducing hospital LOS and charges by 15%, respectively.

## Data Availability

The datasets presented in this article are not readily available because the data are available for purchase from the Healthcare Cost and Utilization Project (HCUP) National Inpatient Sample between January 2002 and December 2021. Others can access the data by contacting HCUP through the HCUP Central Distributor (https://hcup-us.ahrq.gov/tech_assist/centdist.jsp) and purchasing the relevant years of data. This is how the authors accessed these data; the authors did not have any special access privileges others would not have. Requests to access the datasets should be directed to https://hcup-us.ahrq.gov/tech_assist/centdist.jsp.
