# Supplementary material for: Palliative care impact on hospital utilization and trends among lung cancer admissions with a high risk of mortality in U.S. hospitals
Source: Front Health Serv. 2026 Jul 13;6:1801511. doi: 10.3389/frhs.2026.1801511 (PMC13402468; doi:10.3389/frhs.2026.1801511)
Supplement: Supplementary file 1 [file Supplementaryfile1.docx]

Q1. Authors ensured it is correct and that no meaning was lost in the process.

Q2. Confirm that article title is correct.

Q3. All author names are correct.

Q4. No further link is provided at this time.

Q5. No further information at this time.

Q6. State abbreviation affiliations of 2 - NV; 3- CA; 4 - CA; 6 - NV; 7 - NV; 8 - NV; 9 -NV; 10 - NV

Q7. Confirm authors' affiliations

Q8. Confirm email address of corresponding author

Q9. It conveys the intended meaning.

Q10. Confirm the keywords.

Q11. Confirm section headers.

Q12. Confirm all equations and special characters.

Q13. It conveys the intended meaning.

Q14. It conveys the intended meaning.

Q15. It means that only discharge-level values were weighted.

Q16. It conveys the intended meaning.

Q17. It conveys the intended meaning.

Q18. It conveys the intended meaning.

Q19. Keep "discrete" because it is cited from previous studies as same term was used consistently.

Q20. Year 2024

Q21. It conveys the intended meaning.

Q22. Reword by "primary care providers' discussion and documentation of ACP."

Q23. It conveys the intended meaning.

Q24. It conveys the intended meaning.

Q25. It conveys the intended meaning.

Q26. It conveys the intended meaning.

Q27. Confirm this statement is accurate.

Q28. Confirm this statement is accurate.

Q29. Confirm this statement is accurate.

Q30. Confirm this statement is accurate.

Q31. Confirm this statement is accurate.

Q32. Confirm this statement is accurate.

Q33. Confirm references are accurate.

Q34. #11 reference author - Agency for Healthcare Research and Quality; #15 - Office of the Federal Register.

Q35. #14 reference's 6th author -Montgomery L et al; #34 reference's 6th author - Carson J et al.

Q36. No DOI for reference #15.

Q37. Caption of figure 1: Interrupted time series of palliative care utilization among high-risk mortality lung cancer admission

Q38. Authors own original copyright by these charges are paid by the authors.

Q39. All the figures, tables, and captions are correct.

Q40. In the sentence “Tables 2 presents multivariate regression analysis results of impact of PC utilization on LOS and hospital charges.”, this sentence should be corrected by “Tables 2 and 3 present multivariate regression analysis results of impact of PC utilization on LOS and hospital charges, respectively.”

Q41. Confirm below statement is correct.
